# Silicone Wristbands for Measuring Human Exposure to Organic Chemicals: Uses and Benefits for Human Biological Research

**DOI:** 10.1002/ajhb.70187

**Published:** 2026-02-19

**Authors:** Mecca E. Howe, Andrea S. Wiley, Yaw Edu Essandoh, Marta Venier

**Affiliations:** ^1^ Department of Anthropology Indiana University Bloomington USA; ^2^ Urban Institute at the University of North Carolina Charlotte USA; ^3^ O'Neill School of Public and Environmental Affairs Indiana University Bloomington USA

**Keywords:** chemical exposure, human biology, noninvasive, passive sampler, personal sampling device, silicone wristband samplers

## Abstract

Increasing human exposure to environmental contaminants is a growing concern and has become an important factor within human biological variation and health outcomes. Yet, traditional exposure assessment methods are often limited in their ability to capture the complexity and variation of chemical exposure, or are invasive, costly, and challenging to apply in field‐based research. Here, we introduce silicone wristbands as an innovative and noninvasive tool for measuring personal passive chemical exposure and highlight opportunities for their use in human biological research. The wristbands sequester organic chemicals across multiple media (e.g., air, water, dust) and capture both inhalation and dermal absorption. We describe how they work, how to deploy them in the field, how to extract and analyze the chemical composition, and their methodological advantages for human biological research. A case study assessing exposure to flame retardants and the relationship to body size among girls in Costa Rica demonstrates the application for human biological research in a tropical and remote setting. We argue that wristbands provide a noninvasive method for assessing individual exposomes and understanding how environments are embodied and become a meaningful axis of human biological variation. Additionally, they motivate interdisciplinary, ethical, and community‐engaged research in diverse and hard‐to‐reach populations, aligning with future directions of the field of human biology.

As human biologists, our research largely centers around the intersections of environments and human biology, and aims to understand the determinants of biological variation and their relationship to a range of biological outcomes. Today, exposure to toxic synthetic chemicals is a pervasive environmental challenge, and pollutants have become nearly ubiquitous, even in remote areas, due to increased utilization and global distillation (Sadler and Connell 2012).

Around 1700 new chemicals are introduced to the global market each year (Department of Toxic Substances Control, S. of C., n.d.; European Environment Agency 2019; Muir et al. 2023). Many of these chemicals are bioavailable, meaning they can transport across cellular membranes and have physiological effects. A large portion of synthetic organic chemicals are fat‐soluble (i.e., lipophilic), allowing them to accumulate in the adipose tissue of animals and humans (Gore et al. 2015; Mustieles and Arrebola 2020). Substantial evidence shows that these chemicals influence human physiology, both directly through toxicity and indirectly by influencing hormonal and metabolic activity (Gore et al. 2015). At least 800 chemicals have been classified as endocrine disruptors (European Environment Agency 2020), meaning they interfere with hormonal processes. Endocrine disruption can contribute to hormonal disorders, variation in fecundity and reproductive outcomes, as well as metabolic diseases and cancer risk (Gore et al. 2015). Some chemicals have also been connected to neurological diseases and cognitive impairments (Gore et al. 2015).

Many of the biological outcomes studied among human biologists—such as the timing of life history events, reproductive outcomes, body composition, and disease risk—are driven by or related to hormones. Thus, incorporating chemical exposure into our analyses provides a valuable lens for understanding how environments influence health, or are “embodied” (Krieger 2005), and lead to human biological variation.^1^ Uncovering these associations is increasingly important as synthetic chemicals become progressively prevalent in human ecologies, are environmentally and biologically persistent, disproportionately impact vulnerable populations, and are of growing concern as escalating natural disasters intensify the gravity of exposure.

As part of the methodological Toolkit series and Over the Horizons special issue, we introduce silicone wristbands, a recently developed and noninvasive tool for measuring individual exposure to chemicals, and explain the advantages and opportunities for human biological research among diverse populations and settings.

## 1 Traditional Methods and Limitations

Humans are exposed to toxic chemicals through a variety of media: air, water, dust, food, and direct contact with products such as personal care products, furniture, and electronics. In addition, we are exposed to a cocktail of chemicals that can interact in agonistic, antagonistic, or synergistic ways (Dixon et al. 2020). As a result, recent shifts in environmental research have moved beyond studying isolated or a few chemicals toward assessing complex chemical mixtures that more accurately reflect the reality of human exposure. This direction supports emerging conceptual models emphasizing the importance of total exposure throughout one's lifetime, referred to as one's *exposome*, within health and disease outcomes (Lioy and Rappaport 2011; Wild 2012). As more chemicals are classified as emerging concerns, it is critical to understand the cumulative effects of total chemical load and mixtures—key components of an individual's exposome—on human biology and disease risk. However, our understanding remains limited, as traditional methods have been inadequate in capturing individual‐level exposure to complex chemical mixtures.

Much of our current knowledge about human environmental exposure to exogenous contaminants comes from stationary environmental sampling methods such as air monitors, water samples, and dust samples, as well as biological sampling methods. While easy to implement and affordable, stationary environmental samplers cannot provide measures of individual‐level exposure. They often underestimate concentrations (Anderson 2019), are limited to one exposure medium (e.g., air), and only measure concentrations in the nearby space. Furthermore, they miss important intra‐individual exposure variation *within* a space or area (Anderson 2019; Donald et al. 2016; Young 2023).

Personal active samplers have attempted to better measure individual‐level exposure. Active samplers encompass participants carrying air pump monitors with them and are effective at capturing cumulative exposure across different spaces (Anderson et al. 2017). However, they are cumbersome, making them difficult to wear during certain activities (e.g., recreation, sleep), and measure only airborne exposure.

At the other end of the spectrum, researchers interested in the relationship between chemicals and health have relied on measuring chemicals in biological samples such as urine, serum, feces, breast milk, hair, and saliva.^2^ These methods are invasive and can be inappropriate for some sample populations (e.g., children, individuals with religious or cultural taboos surrounding biomarkers). They often require lengthy processes of research permits and approvals, especially if samples are to cross international borders. There are also several ethical dilemmas associated with biological samples (i.e., biosamples) and biobanking, particularly among vulnerable groups and those who have historically experienced exploitation and extraction by the scientific community (Moodley et al. 2014; Turner et al. 2018; Virani and Longstaff 2015). Biosampling is also costly and can be difficult to achieve in remote field settings. Furthermore, chemicals have different internal half‐lives and molecular weights that impact the ability to measure them in biomarkers as well as the amount measured (Anderson et al. 2017; Samon, Hammel, et al. 2022).^3^ Thus, biosampling is limited for measuring chemicals that metabolize quickly or have multiple metabolites. Estimates will also vary based on individual physiological factors (e.g., metabolism, age, and health status) (Dixon et al. 2020; Samon, Hammel, et al. 2022), making interpretations of results and comparisons within and across samples challenging.

## 2 Silicone Wristbands

**FIGURE 1:**
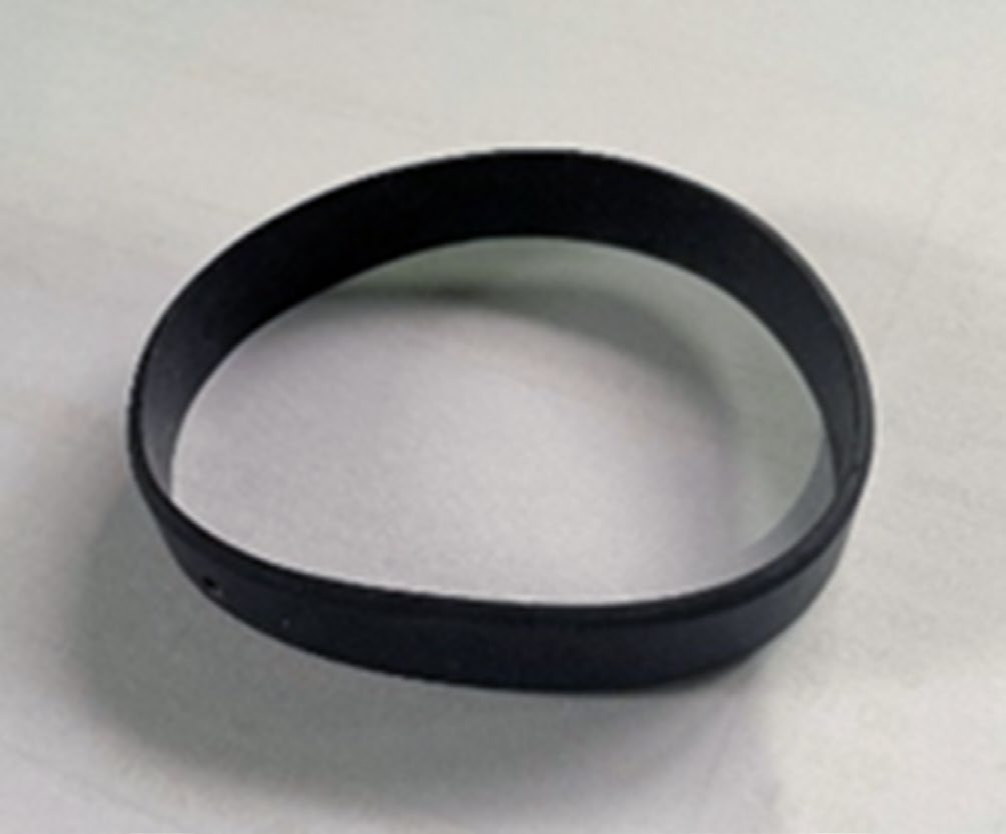
Silicone wristband sampler.

To address the limitations of traditional sampling methods and improve the feasibility of capturing personal exposure to complex chemical mixtures using noninvasive methods, researchers at Oregon State University developed a methodology that uses silicone wristbands as personal passive samplers (O'Connell et al. 2014) (Figure 1). Silicone is highly lipophilic and sequesters organic volatile and semi‐volatile organic chemicals it encounters from water, air, dust, and direct contact (Anderson 2019; Dixon et al. 2020). Participants wear the wristbands consistently for a fixed period that is determined by the chemicals of interest, their affinity, and the goals of the study (e.g., 1–7 days; during work hours or continuously) (O'Connell et al. 2022; Samon et al. 2024; Quintana et al. 2019). The chemicals are then extracted from the wristbands, and extracts are cleaned, concentrated, and analyzed with various analytical techniques; the most common of which include liquid or gas chromatography combined with mass spectrometry (LC/MS and GC/MS). The mass spectra (histograms of abundance of the fragment ions' mass‐to‐charge ratio) are analyzed using a reference database (i.e., mass spectral library) of known compounds to confirm the chemical and its concentration.

Concentrations of chemicals on the wristbands are to be considered time‐weighted averages (from the linear uptake phase), unless the wristband was worn for enough time to reach equilibrium, a condition in which the chemical is not absorbed any further (O'Connell et al. 2022; Samon, Hammel, et al. 2022). In that scenario, the concentration is no longer a time‐weighted average. Although the time to reach equilibrium has been estimated for some chemicals (see: Frederiksen et al. 2022 (polychlorinated biphenyls (PCBs)); Okeme et al. 2018 (phthalates and organophosphate esters (OPEs)); Shoeib and Harner 2002 (PCBs and polychlorinated naphthalene homologue groups); Tromp et al. 2019 (PCBs, polycyclic aromatic hydrocarbons (PAH), musk compounds, phthalates, pesticides, organophosphate esters (OPEs)); Vorkamp et al. 2016 (PCBs)), the majority of studies deploy wristbands for 1–7 days, which is usually insufficient to reach equilibrium. In general, concentrations on the wristband should be corrected for deployment time (e.g., ng/g/day) when comparing with other samples and studies with different wear durations.

The silicone wristband method has been shown to integrate inhalation and dermal exposure (Venier et al. 2018; Hammel et al. 2025). However, they are limited in their ability to estimate exposure through ingestion. Studies have shown that at least for some groups of compounds the levels of chemicals on the wristbands correlate with concentrations measured in urine (Dixon et al. 2018; Gibson et al. 2019; Quintana et al. 2019; Veludo et al. 2024; Wise et al. 2021; Xie et al. 2021), serum (Hammel et al. 2018), plasma (Nguyen et al. 2020), dust (Xie et al. 2021), hand wipes (Hammel et al. 2025; Xie et al. 2021), and air samplers (Dixon et al. 2018; Hammel et al. 2025; Hendryx et al. 2020; Frederiksen et al. 2022); and they may provide better estimates compared to personal air monitors (Wang, Romanak, Stubbings, et al. 2019) and hand wipes (Gibson et al. 2019; Hammel et al. 2016; Wang, Romanak, Stubbings, et al. 2019).

A significant benefit of the tool is that many sequestered chemicals remain relatively stable in the silicone wristbands at ambient temperatures as high as 30°C for up to 1 month, depending on the chemical (Anderson et al. 2017). However, it remains best protocol to freeze the wristbands as soon as possible for optimal stability, with −20°C being associated with the longest stability periods of up to 6 months (Anderson et al. 2017).

To date, there are at least 60 peer‐reviewed. articles featuring the new technology (Bramer et al. 2024), which can measure more than 1500 organic chemicals with a wide range of physiochemical properties including PAHs, oxygenated and nitrated polycyclic aromatic hydrocarbons (OPAHs and NPAHs), PCBs, fragrances, flame retardants (e.g., polybrominated diphenyl ethers (PBDEs)), industrial contaminants, phthalates, personal care products, pharmaceuticals, plasticizers, pesticides, and volatile organic compounds (VOCs) (see the useful resources box for a full list).

The wristbands have been used successfully in a wide range of countries^4^ in different ecological zones and among various participant groups, including children, adolescents, pregnant persons, office workers, roofers, farmers, fishermen, firefighters, nail salon workers, coal miners, demolition workers, and waste workers (Akmermer and Demirtepe 2025; Hammel and Frederiksen 2024; Essandoh et al. 2025; Mohammed et al. 2023; Running et al. 2023; Samon, Hammel, et al. 2022; Santiago et al. 2021; Veludo et al. 2024; Young et al. 2023). They have also provided insights into exposures to toxic waste related to natural disasters such as Hurricane Harvey (Samon, Rohlman, et al. 2022; Samon, Rohlman, et al. 2023).

Studies have used silicone wristbands to measure personal chemical exposures and their relationships with human biological and health outcomes, including DNA damage, cholinesterase inhibition, hormonal activity, menstrual cycle patterns, inflammation, lung function, wheezing, metabolism, BMI, and parity (see Table 1). While these studies evaluated different types of chemical classes, few assessed the contributions of the chemical mixtures or the sum of all chemicals measured to the variation in the biological/health outcome of interest.

**TABLE 1:** Investigations using wristband samplers to explore associations between chemical exposure and human biology/health outcomes.

Authors	Date	Sample	Site(s)	Chemicals measured	Findings
Vidi et al.	2017	10 children (7–9 years)	North Carolina, United States	Pesticides	*DNA damage* in the papilla region significantly correlated (+) with a higher number of pesticide detections
Hardos et al.	2020	76 aircraft maintenance workers	Georgia, Utah, and Arizona, United States	Organophosphate esters (OPEs)	No significant association between exposure and *cholinesterase inhibition*
Kassotis et al.	2020	36 adults with papillary thyroid cancer (PTC) and 36 adults without a history of thyroid disease (control)	North Carolina, United States	Brominated flame retardants (BFRs), OPEs, pesticides, and phthalates	Wristbands can be used to measure nuclear receptor bioactivities. Chemical concentrations were not significantly associated with odds of PTC, but individual chemicals correlated (+) with *thyroid hormone receptor antagonism* in the bioactivity wristband analyses.
Wang et al.	2019b	101 adults, both male and female	Central Appalachia, United States	Novel brominated flame retardants (nBFRs), polybrominated diphenyl ethers (PBDEs, flame retardants), and organophosphate flame retardants (OPFRs)	Specific individual flame retardants were significantly (+ and −) correlated with *thyroid hormone levels*, including free thyroxine (FT4), free triiodothyronine (FT3), and thyroid‐stimulating hormone (TSH)
Andersen et al.^a^	2021	79 Air Force ground crew employees	Denmark	Polycyclic aromatic hydrocarbons (PAHs) and OPEs	No significant association between exposure and inflammation, genetic damage, or lung function
Doherty et al.	2021	177 pregnant women	New Hampshire, United States	Chemicals in personal care products, chemicals in consumer products, pesticides, flame retardants, PAHs, and pharmaceuticals	Differences in total chemical exposure were not significantly associated with *metabolic pathway enrichment*, but individual chemicals (e.g., tonalide, benzyl salicylate) were associated with specific amino acids and variation in ammonia recycling, glutamate metabolism, and phenylalanine and tyrosine metabolism.
Samon et al.	2023	92 pregnant women	New York City, New York, United States	OPEs, BFRs, PCBs, pesticides, and phthalates	Pre‐pregnancy *BMI* was significantly associated (+) with the flame retardants BDE 28/33 and BDE 209 and the plasticizers DEHT and EHDPP. *Multiparity* was significantly associated with lower exposure to the flame retardants TCPP and TDCPP but higher exposure to DEHT.
Young et al.	2023	243 office workers	United States, United Kingdom, China, India	OPEs, phthalates, PAHs, pesticides, BFRs, PCBs	Chemical mixtures extracted from the wristbands were *hormonally bioactive* in human cell assays, including antagonization of thyroid hormone receptor activity, antagonization of androgen receptor activity, and agonization of estrogen receptor activity.
Baker et al.	2024	55 children (5–9 years)	Washington, United States	Flame retardants: BFRs, OPEs, polychlorinated biphenyls (PCBs), polycyclic aromatic hydrocarbons (PAHs), phthalates, and pesticides	Higher exposure to PAH chrysene, the pesticide cis‐permethrin, and the phthalate di‐isononyl (DINP) associated with an increased risk of *child wheeze*.
Howe	2024	54 children (8–16 years)	Sarapiquí, Costa Rica	Pesticides and flame retardants	∑OCPs associated with *later menarche,* and ∑fungicides and azoxystrobin were associated with *earlier menarche*
Soni et al.	2025	105 children (8–11 years)	North Carolina, United States	Pesticides	Potential influence of exposure to organochlorines and pyrethroids on *cognitive scores*

*Note:* See Hamzai et al. (2022), Samon, Hammel, et al. (2022), Running et al. (2023), and Wacławik et al. (2022) for more comprehensive lists of studies utilizing silicone wristbands, including those not assessing health outcomes.

^a^ In Andersen et al. (2021), participants wore silicone bands as pendants on their clothes and not on their wrists.

### 2.1 Field Methods

Any commercial silicone wristband can be used, as long as it is pure silicone and cleaned to remove manufacturing contaminants before deployment. Cleaned wristbands are individually wrapped in aluminum foil and labeled. Once prepped, the aluminum‐wrapped wristbands can be transported in sealed plastic bags at ambient temperature to the field site and stored until use. It is recommended that child‐sized wristbands be used for children and adults with smaller wrists. Participants can be directed to put the wristband on themselves, or investigators may place the wristband using sterilized disposable gloves. Participants should wear the wristband^5^ consistently, including while bathing, hand washing, participating in activities, and sleeping, for a minimum of 3 days. The exact allocation date and time must be documented to calculate the wear duration. Researchers, if present, should also ensure the wristband fits comfortably and is neither too tight nor too loose.

Cleaned field blanks should be administered, when possible, to correct for background ambient contamination and used as quality controls in the post‐deployment analysis stage. Field blanks will go through the same cleaning and extraction protocol as the wristbands worn but are not deployed among participants. Field blanks are momentarily opened in each data collection setting then reclosed and stored until lab analysis.

**FIGURE 2:**
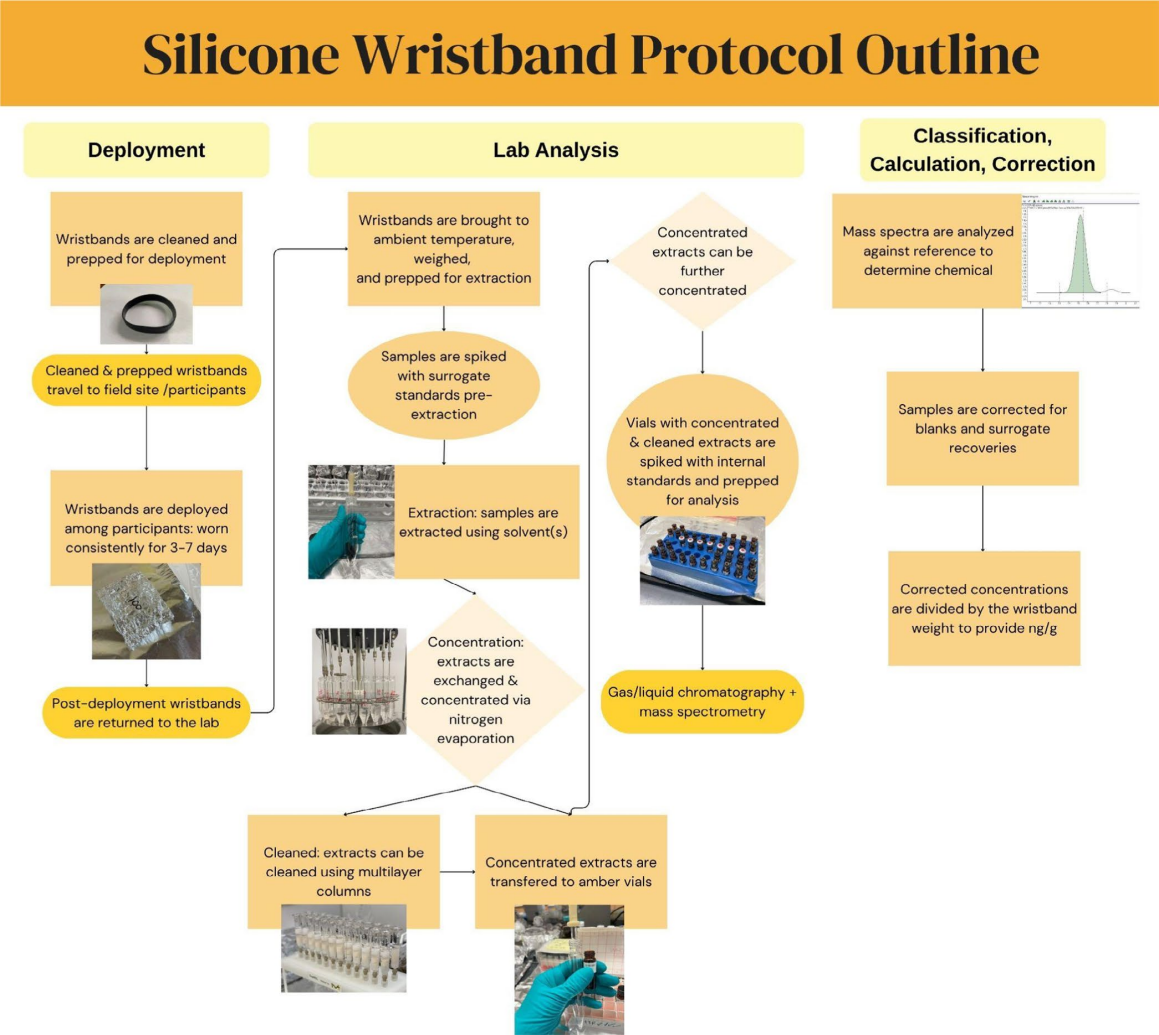
Simplified workflow of silicone wristband sampler protocol used in the case study, including field deployment, laboratory analysis, and calculations and corrections procedures. A full protocol for the case study can be provided upon request.

After the participant has worn the wristband for the determined duration, they personally remove it and place it back in its original aluminum wrapper (see an example in Figure 2). Either the researcher (if present) or the participant will document the exact date and time of wristband removal. The aluminum wrapped wristbands are placed in sealed plastic bags and are either stored until transport if researchers are present or mailed by the participant to the designated lab (with clear instructions provided). The wristbands can be transported at room temperature and then stored at 4°C or colder (Anderson 2019) (−20°C is most common for storing).

### 2.2 Laboratory Methods

Laboratory protocols will vary depending on the class of chemicals of interest. Examples of protocols can be found in: Donald et al. (2016); O'Connell et al. (2014); Romanak et al. (2019); Wang et al. (2019); Wang et al. (2019); Wang et al. (2020); Essandoh et al. (2025). Some steps will overlap among protocols, and a brief description is provided here. Wristbands are weighed, cut into subsections, some of which can be saved for future analyses, cut into smaller pieces to fit into smaller test tubes, and spiked with surrogate standards (see glossary in the Appendix S1) to later be used for quality control to test method efficiency. Next, chemicals are extracted from the wristband pieces using a solvent or combination of solvents (e.g., ethyl acetate, 1:1 hexane/acetone) and concentrated using nitrogen evaporation. Some protocols incorporate an additional step here that cleans the extracts using column chromatography and/or acid cleanups earlier in the protocol to remove interferences and unwanted compounds (see Wang, Romanak, Stubbings, et al. 2019; Wang, Romanak, Hendryx, et al. 2019; Romanak et al. 2019). Concentrated extracts are spiked with internal standards, which are used to quantify the chemicals of interest.

The spiked extracts are measured using instrumental analytical techniques, most of which employ gas or liquid chromatography (depending on the chemical class you were measuring) coupled with mass spectrometry. Quality controls include field blanks, travel blanks, and processing blanks (i.e., laboratory blanks) as well as surrogate standards and matrix spike recoveries. Before calculating the final concentration of each chemical (by dividing the mass measured in each sample by the weight of the wristband used), samples might need to be corrected for blanks if levels in blanks exceed a certain threshold (e.g., 10%–30% of the mass in field‐deployed wristbands). If levels of the chemicals of interest are high in blanks, their levels are subtracted from those in samples on a mass basis.

## 3 Key Advantages of Wristbands

- *Bioavailability*: The wristband samplers act as lipophilic polymers mimicking biological cellular uptake through diffusion (Anderson et al. 2017; Dixon et al. 2020). Thus, they measure chemicals in the environment that have the potential to cross the cellular barrier (Anderson et al. 2017; Dixon et al. 2020). This allows measures to be applied to understand implications on human health.
- *Multiple analyses capabilities*: Investigators can run analyses for multiple different types of chemicals with one to few extractions, offering an opportunity to evaluate chemical mixtures and the total exposure concentrations for all target chemicals (Romanak et al. 2019; Wacławik et al. 2022).
- *Time‐weighted average*: The wristband provides an average chemical concentration over the wear period and can capture very low exposure levels. This time‐weighted average provides a better estimate of one's true chemical uptake compared to conventional sampling methods that only provide a snapshot and need multiple measures over time to evaluate long‐term exposure.
- *Individual‐level data*: The ability to measure personal exposure allows investigators to assess intra‐individual variation, including within homes, neighborhoods, cities, and regions.
- *Ease of participation and high receptiveness*: The wristband samplers are comfortable, do not interfere with activities, and are noninvasive. Studies document high receptiveness to the tool and participation requirements, contributing to easier recruitment and access to various participant groups and higher participation rates (Chanti‐Ketterl et al. 2025; Samon, Rohlman, et al. 2022; Wendee 2018). Furthermore, the ability to take part in the data collection process (i.e., citizen science) has been associated with greater engagement (Rohlman et al. 2022).
- *Simple protocol and high adherence*: The sampler instructions are easy to follow, and the participant burden is low. This results in high protocol adherence among participants and, subsequently, strong methodological integrity and implementation accuracy. In addition, because of the protocol's simplicity, the samplers can be administered and returned through the mail, making them useful for studies in which investigators cannot be present (e.g., during a global pandemic, Baker et al. 2024).
- *Easy transport*: The wristbands are durable, compact, and lightweight, making them easy to transport in large quantities. Since they don't require immediate refrigeration (Anderson et al. 2017), transportation is simpler and more cost‐effective.
- *Easier approval process*: Because they are not biological samples, they require less red tape and time for permits and often do not require transport permits. The sampling method can be determined to have minimal to no risk for Institutional Review Board (IRB) proposals.
- *Field site feasibility*: Since the wristband samplers can be transported and stored at ambient temperatures for 1–3 months (Anderson et al. 2017), they can be shipped long distances and used in virtually any field setting, including remote contexts without access to laboratories/freezers. However, it remains best practice to refrigerate or freeze wristbands when possible to extend stability time.
- *Microenvironments*: To measure and understand exposure from specific settings, the wristbands can be isolated to microenvironments such as office buildings, homes, and occupational settings (e.g., see Akmermer and Demirtepe 2025; Andersen et al. 2021; Nguyen et al. 2020; Young et al. 2023). Participants can be directed to put on their wristband when they arrive and take it off when they leave. It is important to note, though, that individuals bring chemicals from the external environment into indoor settings, which can influence the exposures detected by the wristbands. To minimize interference from personal care products applied to the skin, wristbands can be worn over clothing or placed as stationary samplers (e.g., Hammel et al. 2025). However, this approach will not account for changes due to hand washing or direct contact with substances via the hands/wrists.
- *Natural disasters*: With climate‐related natural disasters on the rise, passive samplers such as silicone wristbands offer an optimal option for measuring exposure to disaster‐related toxic waste (Samon, Rohlman, et al. 2022; Samon, Rohlman, et al. 2023; Wendee 2018). They are quick to deploy, easy to use, and can engage community members in the data collection and research. This is especially valuable when it is impossible for investigators to access the field site and/or impractical to engage directly with participants (Rohlman et al. 2022; Baker et al. 2024). The samplers can provide critical, location‐specific, and timely data for understanding environmental health risks and associated outcomes during and after such events. As an example, see Samon, Rohlman, et al. (2023), which utilized silicone wristbands to measure exposure to toxins after Hurricane Harvey.
- *Ethical considerations and community engagement*: The wristbands are noninvasive, making them more suitable for vulnerable populations, especially when the use of biomarkers is not culturally appropriate. The method also mitigates ethical questions related to the transportation and storage of biological samples. Furthermore, the analysis provides individual‐level results that can be given back to each participant to show them their personal chemical exposure data. This knowledge sharing allows participants to be better informed about chemicals in their environments and how they may take action to reduce their exposures, if possible. It also engages community members in science and research and shows care, leading to better relationships and improved trust among communities and investigators (Bramer et al. 2024; Reams et al. 2017; Riley et al. 2024; Rohlman et al. 2022, 2019). In the case study described below, participants were beyond grateful to receive their personalized data, and this knowledge sharing led to broader conversations with stakeholders and the co‐development of a community agroecological initiative with a local organization and schools.

## 4 Limitations

- Using wristbands alone will not provide insights into specific exposure routes or location point sources, as the wristbands capture cumulative exposure from a variety of media and spaces. Cumulative exposure also limits the ability to detect high uptake peaks associated with specific exposure events. Therefore, it is difficult to determine whether elevated concentrations reflect steady, ongoing exposure or isolated high‐exposure events.
- The wristbands do not fully account for exposure through ingestion and are not an alternative measure for internal dose. Therefore, exposure estimates from wristband extractions cannot be compared with concentrations measured in biomarkers.
- To date, the wristbands are not applicable for measuring inorganic chemicals (e.g., inorganic metals and gases), which have known biological impacts.
- Results from wristbands may be hard to compare across studies (labs) due to the lack of standardization of protocols for extraction and analysis.
- Endogenous compounds such as hormones can be sequestered by the wristbands from dermal excretion, which may interfere with bioassay analyses if not removed from the extracts before analyses. To avoid dermal interference, wristbands or silicone brooches can be worn on clothing (see Andersen et al. 2021, for an example). Alternatively, the Fresh Air Wristband uses a mechanism for capturing airborne exposure to nonpolar chemicals that is encapsulated (much like the face of a watch) and avoids skin contact (see Lin et al. 2020).

## 5 Case Study: Evaluating Exposure to Flame Retardants and Associations With Growth and Development Among Girls

The case study presented is part of a larger research project that measured exposure to pesticides and brominated flame retardants (FRs) among preadolescent and adolescent girls to evaluate whether socio‐ecological contexts were a determinant of exposure variation and whether exposure variation contributed to differences in growth and development (Howe 2024). Findings related to pesticide exposure are published elsewhere (see Howe et al. 2025). Here, we present data on FRs and body size (weight, height‐for‐age *z*‐scores (HAZ), BMI‐for‐age *z*‐scores (BMI‐Z), and leg length). Brominated FRs have been widely used in consumer products and manufacturing materials, including plastics, foam, electrical and automotive parts, construction materials, rubbers, textiles, fabrics, and furniture (Kodavanti et al. 2022). Brominated FRs are recognized as endocrine disruptors. Studies find associations between brominated FRs and thyroid hormones and function (Kassotis et al. 2020; Schell and Gallo 2010; Schell et al. 2012; Wang et al. 2020; Young et al. 2023), cortisol levels (Ikeda‐Araki et al. 2025; El Kouche et al. 2025; Steiniche et al. 2023), estrogen/androgen activity (Ji et al. 2020), and developmental and reproductive outcomes, including variation in the timing of puberty among both boys and girls (Harley et al. 2017) and ovarian function (Wang et al. 2021). However, few publications assess the connections between FRs and variation in body size or composition, which are largely driven by hormonal pathways, and nearly all previous assessments rely on biological sampling methods (Kim et al. 2014).

### 5.1 Methods

The research was approved by the Indiana University Institutional Review Board, the University of Costa Rica Ethical Scientific Committee (CEC), and the Costa Rica Ministry of Health National Health Research Council (CONIS). Wristbands were purchased from 24hourwristbands.com (n.d.), cleaned, and prepped by the Venier Environmental Chemistry Lab at Indiana University and deployed among 192 girls living in a rural industrial‐agricultural region of Costa Rica. Researchers also collected survey and interview data, including 24‐h dietary recalls, and anthropometric measures. Girls were recruited from local schools, churches, community organizations, word‐of‐mouth, and participants' contacts (i.e., snowball sampling, see Howe 2024, for more details about the sampling strategy). Participants provided informed assent, and parents/legal guardians provided informed consent before participation.

The field methods followed those described above. Due to the wristbands being too large for some children, we used riveting to make a subset of the wristbands smaller, creating two smaller size options (a total of three options, including the original). Before giving participants their wristband samplers, they tried on the various sizes to find the best fit. Then, girls were provided with a wristband of the chosen size, which they wore consistently for at least 72 h (mean wear duration = 4.5 days). Wristbands were collected on an agreed‐upon date and time. At the time of collection, the girls removed their wristband and placed it in their original labeled aluminum foil wrappers. The aluminum‐wrapped wristbands were then placed and sealed in individual Ziplock bags labeled with the participant number, transported in a cooler bag with gel icepacks, and stored in −20°C to −10°C freezers at the Organization for Tropical Studies La Selva Biological Research Station. At the close of fieldwork, the wristbands were shipped to the Venier Environmental Chemistry Lab at Indiana University and stored in −20°C freezers until lab analysis.

For this analysis, we used a subsample of 54 wristbands stratified by age, location, and household to capture individuals of all ages who lived in different socio‐ecological contexts. We tested for 35 brominated FRs (24 polybrominated diphenyl ethers (PBDEs), 10 novel brominated flame retardants (nBFRs), and hexabromocyclododecane (HBCD), included in the sum of nBFRs for analysis). We additionally analyzed five field blanks and six lab blanks for quality control.

Wristband halves were extracted and analyzed in batches of 10–12. The detailed steps for the laboratory methods are described in Wang et al. (2020) and Essandoh et al. (2025). In short, we used solvent extraction and multicolumn cleaning. FRs were analyzed using gas chromatography/mass spectrometry with an Agilent 7890B series GC coupled to an Agilent 5977B MS. Mass spectra were assessed using the Agilent MassHunter software by the Venier Lab team.

Average surrogate recoveries were 64% for BDE‐77, 100% for BDE‐166, and 88% for 13C12‐209. The mean concentrations detected in blanks, along with detection frequencies, are provided in Table A1 in Appendix S1. pTBX, PBBZ, PBEB, BDE‐49, BDE‐47, and BDE‐100 were not detected in any blanks. Percent of blanks for the samplers ranged from 0.2% to 36%, with an average of 9%. If blank concentrations fell within 10%–35% of the measured sample, the blank concentration was subtracted from wristband samples on a mass basis. If the mass detected in the blank was more than 35% of that in the actual wristband, the value was replaced with half of the instrument detection limit. The mass of chemicals detected on the wristbands was converted to ng/g based on the weights of the wristband halves.

The sum of all PBDEs, nBFRs, and all FRs detected in each wristband was calculated to create a ∑PBDEs, ∑nBFRs, and ∑FRs variable for hypothesis testing. The logs for ∑PBDEs, ∑nBFRs, ∑FRs, and the most frequently detected FRs were calculated and used for all hypothesis tests to achieve normal distribution. Using Stata/SE 19, we first conducted univariate regression analyses to test significant associations between independent variables with known connections to body size and composition—household income, household size, having a self‐reported health condition (not including allergies), and daily caloric consumption—controlling for age (alpha = 0.05). Due to limited variation in household income, income was dichotomized into < and ≥ sample median. Independent variables with significant associations with anthropometrics were controlled for, along with age, in models assessing associations between log‐transformed FRs and anthropometrics.

### 5.2 Results

The mean age of the participants was 12.6 years. Around 43% lived in households with monthly incomes below the sample median ($405), and 37% reported a health condition (e.g., asthma, skin disorder, mental health condition, gastrointestinal issues, migraines, sinusitis). None reported a current infectious disease. Eight girls (15%) had BMI‐for‐age *z*‐scores (BMI‐Z) considered “overweight”, and two (4%) had BMI‐Z deemed “obese” (WHO n.d.). None were considered “underweight”, and only one fell into the category of “stunting” based on height‐for‐age *z* score (HAZ) (WHO 2018).

Detection frequencies, mean, and median concentrations, corrected for blanks, for compounds detected in at least 25% of sample wristbands are presented in Table A1 in Appendix S1. On average, 12 FRs were detected in the sample wristbands, with mean concentrations ranging from 0.002 to 24.4 ng/g. Thirteen PBDEs were detected in more than 25% of wristbands, and PBDEs with the highest detection frequencies included BDE‐197 (78%), BDE‐209 (78%), BDE‐47 (76%), and BDE‐207 (76%). Five nBFRs were detected in at least 25% of samplers, and decabromodiphenyl ethane (DBDPE) (91%) and DPs (syn‐DP and anti‐DP) (89%) had the highest detection frequencies. FRs with the highest mean concentrations after blank corrections included BEHTBP, EHTBB, and BDE‐209. These three compounds are the active ingredients of the most used commercial mixtures in recent years, as opposed to legacy mixtures that were removed from the market (i.e., PenaBDE, which was made up mostly of BDE47, 99, and 100). This find reflects a relatively fresh source of FRs in this area.

Household income, self‐reported health condition, and daily caloric consumption were not predictors of weight, HAZ, BMI‐Z, or leg length when controlling for age (see Table A2 in Appendix S1). Household size was significantly associated with weight. Participants living in a household with four or five members had lower weights than those from households of two to three (*b* = −6.4, *p* = 0.037; *b* = −7.1, *p* = 0.025, respectively). Household size was not associated with HAZ, BMI‐Z, or leg length.

Log‐transformed ∑FRs, ∑PBDEs, and ∑nBFRs were not associated with weight (controlling for age and household size), HAZ, or leg length, but ∑PBDEs and ∑FRs were positively associated with BMI‐Z (*b* = 0.32, *p* = 0.045; *b* = 0.26, *p* = 0.046, respectively) (Table A2 in Appendix S1). Of the FRs with the highest detection frequency, log‐transformed BDE‐147, BDE‐197, BDE‐209, DPs, and DBDPE were not predictive of weight, HAZ, BMI‐Z, or leg length. BDE‐207 was positively associated with HAZ (*b* = 0.43, *p* = 0.036), but not significantly related to weight, BMI‐Z, or leg length.

### 5.3 Conclusions

The case study documents exposure to a wide range of FRs among children and adolescents from an understudied rural population, and higher exposure concentrations of log‐transformed summed PBDEs and FRs, specifically, were associated with higher BMI *z*‐scores. As nBFRs were not related to BMI‐Z variation, it is likely that PBDEs are driving the association between total sum FRs and BMI among the sample. However, no single FR was predictive of BMI, implying that cumulative exposure may be a more appropriate indicator for measuring biological impacts. Surprisingly, most household characteristics, including income and daily caloric intake, were not significantly associated with body size or composition. This may be the result of the small sample size and reliance on a single 24‐h dietary recall.

Studies have found associations between exposure to FRs and impacts on thyroid hormone concentrations and function, cortisol levels, and sex steroids—which have important roles in regulating metabolism (Kassotis et al. 2020; Levy and Bribiescas 2023; Schell et al. 2012; Steiniche et al. 2023; Ji et al. 2020; Wang et al. 2020; Young et al. 2023; Zhang et al. 2023). The relationship between FRs and thyroid hormones remains inconclusive, as some studies find a positive effect while others document suppressant impacts (Kassotis et al. 2020; Schell et al. 2012; Wang, Romanak, Hendryx, et al. 2019; Yeshoua et al. 2024). In studies among adolescents from the Akwesasne Nation with a high prevalence of overweight and obesity, PCB flame retardants were associated with higher thyroid‐stimulating hormone (TSH) and reduced free thyroxin (FT4) (Schell and Gallo 2010). Higher TSH is commonly observed among adolescents with BMI *z*‐scores indicative of overweight and obesity (Stáníková et al. 2023; Priya et al. 2020). Other investigations find FR exposure associates with lower cortisol levels in peripubertal children and adults (Ikeda‐Araki et al. 2025; El Kouche et al. 2025), and lower circulating cortisol has been linked with higher BMIs and overweight status among adolescents (Ruttle et al. 2013). Thus, endocrine disruption may account for the connection between higher FR exposure concentrations and higher BMIs in this sample.

## 6 Methodological Takeaways

Participants were highly receptive to and intrigued by the wristband samplers.^6^ Only one participant lost her wristband. They were also appreciative of receiving personalized results that described each chemical detected in their wristband, its concentration, uses, and associated health risks. The full study findings were provided to all participants, including those whose wristbands were not analyzed, via one‐page summaries, as well as provided to community organizations, schools, and the Ministries.

The Costa Rican Ministry of Health's National Committee of Health Research expressed enthusiasm and appreciation for the method's noninvasive nature. The wristbands also made it easier to get IRB approval, research permits, and conduct research without the hiring of medical practitioners (which we were told is required to collect most biomarkers in Costa Rica). Furthermore, no special permits were needed to ship the wristbands out of the country. We were fortunate to have access to freezers at the biological station, but because the wristbands did not have to be kept at below freezing temperatures, we did not worry about them in transport or when the hot external climate made it difficult for the freezers to stay below −10°C. Notably, the laboratory analysis is costly and time‐consuming, partly due to the large learning curve but also because of the limitation of running 10–12 extractions at one time and the use of three different post‐extraction techniques needed to measure the different chemical classes.^7^ It was important to collaborate with trained and experienced chemists for the mass spectrometry, mass spectrum analyses, and quantifications, as these entail niche knowledge and training.

## 7 Theoretical and Research Applications

- *Embodiment*: The wristband samplers provide a noninvasive avenue for understanding how one's external environment “gets under the skin” (Krieger 2005). It allows us to measure complete exposure to biologically active chemicals. Using wristband data and association studies, we can explore relationships between chemical exposure and biological and health variables, which are of particular value when assessed during critical periods of the life course. Additionally, extracts from samplers can be added to bioassays to evaluate real‐time effects, such as in Young et al. (2023), which assessed the chemical mixtures' contributions to hormonal activation (Young et al. 2023). The changes seen in bioassays provide causal evidence of the direct impacts of one's environment on biology.
- *Exposome*: The exposome framework is a new conceptual and methodological approach to understanding how cumulative environmental exposures throughout the life course impact biological and health outcomes. Chemicals are an inevitable part of the broader “exposome”—an individual's total environmental exposure load throughout their life (i.e., accumulative life exposure load)—and therefore should be considered within research that aims to understand the environmental contributions to biological variation and health (Goodrich et al. 2016; Vrijheid 2014; Wild 2012). Silicone wristbands are a valuable tool for this context, as they can sequester more than 1500 organic chemicals. Using the wristbands to measure such a wide range of chemicals can contribute to understanding an individual's exposome during specific life stages. Furthermore, wristbands allow for the evaluation of chemical mixtures and interactions (Dixon et al. 2020), improving our understanding of the complex impacts of environmental chemical exposure on human biology and health.
- *Equity*: The wristbands provide individualized exposure data that can be compared across participants and samples to highlight important variation in who is most exposed and to what. The literature is saturated with evidence that populations of minority, lower‐income, and certain occupational groups are disproportionately exposed to toxic contaminants (Gochfeld and Burger 2011; Payne‐Sturges et al. 2023; Puckrein and Rich 2024). Using wristbands to obtain estimates of total chemical exposures from all participant spaces can contribute invaluable information and arguments around equity and environmental justice.
- *Community‐engaged research*: The ability to measure personal chemical exposure without invasive methods and participant burden creates opportunities for more research, in general, but also builds trust and contributes to community‐engaged research, including among hard‐to‐reach communities. Researchers can empower community members through the provisioning of individualized data and knowledge about the chemicals they are exposed to and their relative health risks. Additionally, the method actively engages participants in the research as citizen scientists in the data collection process. In our experience, the wristbands can make research seem “fun” and accessible, especially to younger participants.
- *Interdisciplinary research*: Laboratory analysis of the wristband samplers requires collaborations with trained chemists. At the same time, social scientists are necessary to provide contexts for understanding the causes of exposure and variation. Human biologists, epidemiologists, and endocrinologists can speak to the physiological effects and health outcomes. Anthropologists, specifically, provide biocultural approaches that encompass both evolutionary and sociocultural factors to understand the impacts that chemical exposures have on populations and communities. Thus, the wristbands motivate interdisciplinary research collaborations as they bridge environmental chemistry with biology and health. As our understanding of the complexity and multifaceted nature of human biology and environmental interactions grows, we need interdisciplinary approaches that can bring together the skills and funding necessary to ask new and more complicated questions.
- *Future directions*: Researchers have also used the wristband samplers as a noninvasive alternative for measuring skin microbiome and the interaction with disease vectors (Roodt et al. 2018; Wooding et al. 2020). Others have altered the protocol slightly to create other forms of wearable samples that are easier for animal studies (e.g., pet tags) or to limit the capture of dermal uptake when solely interested in respiratory uptake (e.g., dog tag necklaces and lapels). With natural disasters on the rise, wristbands can also be valuable in measuring pollution exposure post‐event. More evaluations of chemical mixtures and biological outcomes are warranted.

## 8 Conclusion

A central theme of the Human Biology Association Annual Meetings' Over the Horizon session, in celebration of the association's 50th anniversary, was the urgent need for innovative, accessible, and ethical research tools that can keep pace with the complexities of our changing Anthropocene. Within new ecological realities, humans are exposed to a complex mixture of chemicals that may interact with one another and have outcomes that are dependent on the chemical mixtures, life course stage, and other axes of biological variation. Silicone wristbands offer a promising, noninvasive method for assessing a more comprehensive measure of exposures to organic chemicals and mixtures. They are suitable for new conceptual models and inspire interdisciplinary frameworks that combine exposure research with human evolutionary biology and environmental health. Furthermore, the wristbands minimize participant burden and foster community engagement, exemplifying a broader shift in human biology research toward inclusive, collaborative, and responsive research.

**Useful Resources Box**

Websites:

- Silicone Wristband Personal Monitoring Device: Overview and videos from Oregon State University (Oregon State University, Food Safety and Environmental Stewardship Program n.d.‐a): https://fses.oregonstate.edu/wristbands.
- Chemicals that the wristbands can capture (Oregon State University, Food Safety and Environmental Stewardship Program n.d.‐b): https://fses.oregonstate.edu/methods.
- Gas Chromatography/Mass Spectrometry Introduction (Oregon State University, Unsolved Mysteries of Human Health n.d.): https://unsolvedmysteries.oregonstate.edu/gas‐chromatography‐mass‐spectrometry‐introduction.
- My Exposome, Personal Environmental Monitoring (MyExposome Inc, n.d.): https://www.myexposome.com/#about.
- Mass Spectrum Interpretation (U.S. Department of Commerce, National Institute of Standards and Technology 2022): https://www.nist.gov/video/mass‐spectral‐interpretation‐part‐1.

Book Chapters:

- Dixon et al. (2020).

Talks:

- Anderson (2019).
- Young (2023).

Supplies:

- Wristbands (24HourWristbands.com n.d.): https://24hourwristbands.com/.
- Liquid chromatography coupled with mass spectrometry (Agilent Technologies Inc. 2022): https://www.agilent.com/cs/library/datasheets/public/5991‐6152EN.pdf.
- Gas chromatography coupled with mass spectrometry (Agilent Technologies Inc. 2020): https://www.agilent.com/cs/library/brochures/brochure‐source‐of‐new‐possibilities‐5977B‐gc‐msd‐5991‐7620en‐agilent.pdf.
- Agilent MassHunter (mass spectrometry analysis) software (Agilent Technologies Inc. n.d.): https://www.agilent.com/en/promotions/masshunter‐mass‐spec.

## Author Contributions

**Mecca E. Howe:** conceptualization, data curation, formal analysis, funding acquisition, investigation, visualization, writing‐original draft, writing – reviewing and editing. **Andrea S. Wiley:** conceptualization, funding acquisition, resources, supervision, writing – reviewing and editing. **Yaw Edu Essandoh:** data curation, methodology, software, **Marta Venier:** conceptualization, data curation, funding acquisition, methodology, resources, software, supervision, validation, writing – reviewing and editing.

## Funding

The authors have nothing to report.

## Conflicts of Interest

The authors declare no conflicts of interest.

## Supporting information


**APPENDIX S1:** Supporting information.

## Data Availability

The data that support the findings of this study are available from the corresponding author upon reasonable request.

## Endnotes

^1^
Relatively few human biologists have evaluated the links between chemical exposure and biology, and studies have relied on invasive biological sampling methods. Examples of important strides by human biologists include, but are not limited to, Denham et al. (2005) (chemicals measured in blood samples: lead, mercury, mirex, DDE, HCB, and PCBs; biological variables measured: menarche); Schell and Gallo (2010) (chemicals measured in blood samples: PCB, DDE, HCB, mirex, lead, and mercury; biological variables evaluated: thyroid function and sexual maturation); Vallascas et al. (2013) (chemical measured in hair samples: lead; biological variable evaluated: adipose tissue); and Howland et al. (2020) (chemical measured in urine samples: BPA; biological variables evaluated: estrogen metabolites and thelarche). More recently, Chaney et al. (2025) explored complex chemical clusters from urine and milk samples and their relationships with metabolic pathways (Chaney et al. 2025). Their approach exemplifies a shift in research in this area‐moving beyond the study of isolated or a few chemicals toward assessing complex chemical mixtures that better reflect the reality of human exposure.

^2^
Hand wipes and dermal patches have also been used but are less common in studies interested in biological outcomes.

^3^
For example, using blood serum to measure DEHTP will only estimate about 16% of the ingested dose (Samon, Hammel, et al. 2022).

^4^
Countries where the wristbands have been deployed include Bangladesh, Belgium, Chile, China, Denmark, the Dominican Republic, France, Ghana, Guinea, India, Italy, Japan, Peru, Senegal, South Africa, Uganda, Uruguay, the U.S., and now Costa Rica.

^5^
Some protocols recommend that participants wear the wristband on the wrist of the dominant hand to better capture exposure through daily behaviors often conducted using one's dominant hand, such as applying personal care products, cleaning, writing, eating, grabbing, and holding.

^6^
Some participants had questions about the wristband's ability to listen to them and were reassured before participation that they had no listening devices or any type of digital aspects.

^7^
One fraction was used to analyze FRs, and two fractions were used to measure pesticides—published elsewhere—with two different exchange methods: one using Dichloromethane (DCM) and the other using ethylacetate (EA). See Romanak et al. (2019), for more description.
