# Supplementary material for: Silicone Wristbands for Measuring Human Exposure to Organic Chemicals: Uses and Benefits for Human Biological Research
Source: Am J Hum Biol. 2026 Feb 19;38(2):e70187. doi: 10.1002/ajhb.70187 (PMC12917870; doi:10.1002/ajhb.70187)
Supplement: Supplementary file 1 — APPENDIX S1: Supporting information. [file AJHB-38-e70187-s001.docx]

**Appendix A. Glossary**

Active samplers: sampling devices equipped with a motor that collect air through a filter and a cartridge to capture vapor and particle-bound chemicals.

Concentration: the mass of a chemical analyte per mass or volume of sample.

Extraction: the process of separating chemicals from the sampling media (e.g., silicone wristbands) using solvents.

Exposome: an individual’s total environmental exposure load throughout the life course (i.e., cumulative life exposure load). As a theoretical framework, the exposome considers all lived environments and exposures within the determinants of disease risks and outcomes.

Field blanks: wristbands that are cleaned, travel to the research setting, and are exposed to the ambient but not worn by participants

Internal standards: compounds that are chemically similar to target analytes and added in a known amount to all samples before instrumental analysis to estimate concentrations.

Passive samplers: sampling devices that sequester organic chemicals through diffusion, absorption, and partitioning with the air and/or water.

Pre-deployment blanks (i.e., lab blanks): wristbands that were cleaned through the same process as the samplers but are not deployed. Rather, they stay in the lab and are used for quality control, including contamination of the extraction process and analysis.

Travel blanks - wristbands that are cleaned and travel to the research setting but are not opened. These are useful for studies that use mailing to allocate and receive the wristband samplers in the absence of researchers present.

Surrogate standards: compounds that are chemically similar to target analytes and added in a known amount to all samples at the beginning of the extraction process to assess the method's performance.

**Appendix B. Case Study Table A1**

| **Table A1. Detection frequencies and blank-corrected concentrations (ng/g) for compounds detected in at least 25% of wristband samplers** | | | | | | | | | |
| --- | --- | --- | --- | --- | --- | --- | --- | --- | --- |
|  |  | **Wristbands (n = 54)** | | | | | **Blanks (n = 6)** | | |
| **FR** | **IDL (ng)** | **DF%** | **Mean (ng/g)** | **Median (ng/g)** | **Min (>0), Max** | **Mean % Blank Contribution** | **DF%** | **Mean (ng)** | **Median (ng)** |
| **PBDE** |  |  |  |  |  |  |  |  |  |
| BDE-28 | 0.009 | 35.19 | 0.09 | 0.00 | 0.03 - 1.35 | 3.61 | 33.33 | 0.004 | 0.004 |
| BDE-47 | 0.015 | 75.93 | 1.98 | 1.06 | 0.26 - 17.78 | 0.00 | 0.00 | -- | -- |
| BDE-99 | 0.015 | 62.96 | 1.68 | 0.68 | 0.15 - 14.93 | 0.00 | 16.67 | 0.26 | -- |
| BDE-100 | 0.021 | 27.78 | 0.32 | 0.00 | 0.16 - 3.25 | 0.00 | 0.00 | -- | -- |
| BDE-139 | 0.062 | 26.63 | 0.44 | 0.00 | 0.01 - 9.79 | 36.04 | 83.33 | 0.37 | 0.03 |
| BDE-153 | 0.016 | 44.44 | 0.19 | 0.00 | 0.05 - 1.84 | 4.00 | 16.67 | 0.003 | -- |
| BDE-197 | 0.027 | 77.78 | 0.30 | 0.13 | 0.04 - 1.43 | 5.95 | 50.00 | 0.01 | 0.01 |
| BDE-201 | 0.029 | 40.74 | 0.05 | 0.00 | 0.01 - 1.36 | 19.75 | 50.00 | 0.02 | 0.01 |
| BDE-203 | 0.034 | 44.44 | 0.19 | 0.00 | 0.04 - 1.43 | 9.17 | 50.00 | 0.01 | 0.02 |
| BDE-206 | 0.038 | 68.52 | 0.61 | 0.37 | 0.01 - 6.04 | 8.09 | 83.33 | 0.10 | 0.02 |
| BDE-207 | 0.037 | 75.93 | 0.39 | 0.22 | 0.05 - 3.41 | 7.96 | 50.00 | 0.02 | 0.02 |
| BDE-208 | 0.045 | 57.41 | 0.16 | 0.07 | 0.04 - 2.49 | 8.90 | 66.67 | 0.02 | 0.02 |
| BDE-209 | 0.027 | 77.78 | 4.82 | 1.92 | 0.01 - 84.95 | 3.24 | 66.67 | 0.15 | 0.12 |
| Σ PBDEs | -- | -- | 13.38 | 7.58 | 0.43 - 104.36 | -- | -- | 1.40 | 0.92 |
|  |  |  |  |  |  |  |  |  |  |
| **nBFR** |  |  |  |  |  |  |  |  |  |
| pTBX | 0.013 | 27.78 | 0.04 | 0.00 | 0.01 - 0.46 | 0.00 | 0.00 | -- | -- |
| EHTBB | 0.127 | 55.56 | 5.60 | 0.81 | 0.44 - 153.16 | 12.14 | 16.67 | 0.19 | -- |
| DPs* | syn: 0.020; anti: 0.023 | 88.89 | 0.24 | 0.04 | 0.01 - 2.64 | 12.95 | 66.67 | 0.01 | 0.01 |
| BEHTBP | 0.121 | 55.56 | 24.44 | 8.11 | 0.43 - 342.59 | 0.16 | 16.67 | 0.03 | -- |
| DBDPE | 0.080 | 90.74 | 2.78 | 1.06 | 0.02 - 42.20 | 6.99 | 16.67 | 0.06 | -- |
| ΣnBFRs | -- | -- | 29.61 | 12.25 | 0.49 - 358.39 |  | -- | 0.12 | 0.03 |
| **ΣFRs** | -- | -- | **42.24** | **25.35** | **1.04 - 422.09** |  | -- | 1.51 | 1.18 |
| FR = flame retardant, PBDEs = polybrominated diphenyl ethers, nBFRs = new brominated flame retardants, IDL = instrument detection limit (ng), DF = detection frequency, ng/g = nanograms/grams *DPs = sum of syn-DP and anti-DP, as these represent differing isomer shapes of the same molecule Summary statistics include 0s for samples with no detection. Thus, they include all samples (except for the minimum values, which represent the minimum detection > 0 for the sample). | | | | | | | | | |

**Appendix C. Case Study Table A2**

| **Table A2. Regression results, controlling for age** | | | |  |  |  |  |  |  |
| --- | --- | --- | --- | --- | --- | --- | --- | --- | --- |
|  |  | **Weight** | | **HAZ** | | **BMI-Z** | | **Leg Length** | |
|  | **% of sample** | **b** | **p (CI)** | **b** | **p (CI)** | **b** | **p (CI)** | **b** | **p (CI)** |
| Household Income ≥ median | 57% | -1.87 | 0.43  (-6.6, 2.9) | 0.41 | 0.16 (-0.6, 1.0) | 0.13 | 0.66  (-0.5, 0.7) | -3.16 | 0.05 (-6.4, 0.1) |
| Household Size |  |  |  |  |  |  |  |  |  |
| (base) 2-3 | 28% | -- | -- | -- | -- | -- | -- | -- | -- |
| 4 | 28% | -6.37 | 0.04 (-12.4, -0.4) | -0.19 | 0.62 (-1.0, 0.6) | -0.34 | 0.41 (-1.1, 0.5) | -1.23 | 0.58 (-5.7, 3.2) |
| 5 | 26% | -7.05 | 0.03 (-13.2, -0.9) | -0.15 | 0.70 (-0.9, 0.6) | -0.38 | 0.37 (-1.2, 0.5) | -1.69 | 0.46 (-6.2, 2.9) |
| 6+ | 18% | -5.1 | 0.13 (-11.8, 1.6) | -0.38 | 0.39 (-1.2, 0.5) | -0.35 | 0.45  (-1.3, 0.6) | 0.01 | 1.00 (-5.0, 5.0) |
| Health Condition (yes) | 37% | -0.04 | 1.0  (-5.1, 5.1) | 0.47 | 0.17  (-0.2, 1.2) | 0.23 | 0.49  (-0.4, 0.9) | -1.36 | 0.46 (-5.1, 2.3) |
| Calories (Kcal) | 100% | 0.00 | 0.85 (-0.0, 0.0) | 0 | 0.98 (-0.0, 0.0) | 0.00 | 0.83 (-0.0. 0.0) | 0.00 | 0.16 (-0.0, 0.0) |
| ∑FRs |  | -0.29 | 0.77 (-2.3, 1.7) | 0.09 | 0.50 (-0.17, 0.34) | 0.26 | 0.046 (0.01, 0.5) | -0.99 | 0.16 (-2.4, 0.4) |
| ∑PBDEs |  | -1.31 | 0.29 (-3.8, 1.1) | 0.22 | 0.14 (-0.1, 0.5) | 0.32 | 0.045 (0.01, 0.6) | -0.63 | 0.47 (-2.4, 1.1) |
| ∑nBFRs |  | -0.01 | 0.99 (-1.4, 1.4) | 0.03 | 0.70 -0.1, 0.2) | 0.15 | 0.10 (-0.03, 0.3) | -0.85 | 0.08 (-1.8, 0.1) |
| BDE-47 |  | 1.07 | 0.42 (-1.6, 3.7) | 0.15 | 0.27 (-0.1, 0.4) | 0.10 | 0.52 (-0.2, 0.4) | 1.06 | 0.26 (-0.8, 2.9) |
| BDE-197 |  | -0.3 | 0.82 (-3.1, 2.5) | -0.06 | 0.73 (-0.4, 0.3) | -0.01 | 0.96 (-0.3, 0.3) | 0.62 | 0.50 (-1.2, 2.4) |
| BDE-207 |  | -1.17 | 0.51 (-4.7, 2.4) | 0.43 | 0.036 (0.03, 0.8) | 0.34 | 0.09 (-0.1, 0.7) | 0.69 | 0.53 (-1.5, 2.9) |
| BDE-209 |  | -1.35 | 0.05 (-2.7, -0.01) | 0.05 | 0.60 (-0.1, 0.2) | 0 | 0.99 (-0.2, 0.2) | -0.24 | 0.62 (-1.2, 0.7) |
| DPs |  | -0.09 | 0.92 (-1.9, 1.7) | 0 | 0.97 (-0.2, 0.2) | 0.03 | 0.83 (-0.3, 0.3) | 0.98 | 0.10 (-0.2, 2.2) |
| DBDPE |  | 0.06 | 0.93 (-1.4, 1.6) | 0.05 | 0.62 (-0.1, 0.2) | 0.08 | 0.45 (-0.1, 0.3) | -0.35 | 0.52 (-1.4, 0.7) |
| Log-transformed flame-retardant variables were used for all models. All models controlled for age. All models assessing associations between FR variables (independent) and weight (dependent) controlled for household size.  b = coefficient, p = probability, CI = confidence intervals, HAZ = height for age Z score, BMI-Z = BMI for age Z score, ∑ = sum, FR = flame retardant, PBDEs = polybrominated diphenyl ethers, nBFRs = new brominated flame retardants, DPs = sum of syn-DP and anti-DP as these represent differing isomer shapes of the same molecule In models including household characteristics, base comparison variables included household income < sample median, household size of 2-3, and no health condition reported | | | | | | | | | |

**Appendix D. Full list of FRs tested for**

BDE-15

pTBX

PBBZ

BDE-17

BDE-28

PBEB

HBB

BDE-49

BDE-47

BDE-66

BDE-77 (SS)

BDE-100

BDE-99

EHTBB

BDE-85

BDE-154+BB153

BDE-153

BDE-139

HBCD

BDE-140

BDE-166 (SS)

BDE-183

BDE-181 (IS)

TBE

BEHTBP

syn-DP

BDE-201

BDE-197

anti-DP

BDE-203

BDE-208

BDE-207

BDE-206

BDE-209

13C-BDE-209 (SS)

DBDPE

*SS = surrogate standard, IS = internal standard*
